# An ultrasound-guided no incision banding method for the treatment of arteriovenous fistula high-flow in hemodialysis

**DOI:** 10.1080/0886022X.2023.2222853

**Published:** 2023-06-21

**Authors:** Yanan Wang, Jin Li, Wenjun Liu, Ying Zhang, Qing Li, Fan He

**Affiliations:** a Department of Nephrology, Tongji Hospital of Tongji medical college of Huazhong University of Science and Technology, Wuhan, China; b Department of Hematology and Nephropathy, Xishui People’s Hospital, Huanggang City, China

**Keywords:** Arteriovenous fistula, high-flow vascular access, no-incision banding method, hemodialysis

## Abstract

**Objective:**

High-flow vascular access is one of the serious complications in the maturation and subsequent use of arteriovenous fistula (AVF). We adopted a novel surgical approach named no incision limited ligation indwelling needle assisted- revision (NILLINR) to treat high-flow of the hemodialysis vascular access and ascertained the outcomes by regular follow-up visits.

**Methods:**

This is a retrospective study. 26 hemodialysis patients with symptomatic high-flow access (access flow > 1500 mL/min) were treated with the novel banding method without incision between June 2018 and October 2020. The flow of the brachial artery before and after the restriction was measured by experienced clinicians by using the duplex Doppler ultrasound (DUS). All 26 patients were followed up for up to 1 year. Meanwhile, the brachial artery flow was recorded at 6 months and 1 year after restriction.

**Results:**

Of all 26 patients included in this study, the mean access flow volume decreased from 2196.2 ± 416.9 mL/min (mean ± SD) to 679.2 ± 67.1 mL/min immediately after the operation. During the follow-up, the volume flow of the brachial artery was still within the restricted range at 6 months (mean ± SD, 720.2 ± 164.7 mL/min) and 1 year (mean ± SD, 713.9 ± 173.8 mL/min) after the operation. Meanwhile, the mean duration of the operation is 8.5 ± 3.3 min, and there is no bleeding or rupture.

**Conclusion:**

This novel no-incision limited ligation indwelling needle-assisted revision is a safe, effective, and time-saving option to treat high-flow access.

## Introduction

Patent and functional vascular access are necessary for end-stage renal disease (ESRD) patients undergoing maintenance hemodialysis (MHD). Arteriovenous fistula (AVF), arteriovenous graft (AVG), and central venous catheter (CVC) are common types of dialysis access. Among them, autologous AVF is considered to be superior to the other two methods due to its better patency and fewer complications [1,2]. However, there are still a series of complications in the maturation and subsequent use of autologous AVF, such as access stenosis, thrombosis, and high-flow. Autologous AVF must maintain the pressure and flow within an appropriate range to realize effective hemodialysis. With the aging of AVF, the expansion of vascular access leads to the reduction of vascular resistance, which further results in high flow [3]. High-flow access may induce left ventricular hypertrophy, high-output heart failure, and dialysis-associated steal syndrome (DASS) [4,5]. In addition to traditional open surgery, the minimally invasive limited ligation endoluminal-assisted revision (MILLER) banding procedure has been an optimal treatment alternative for dialysis high-flow [6–8]. Here, we introduce a novel approach named no incision limited ligation indwelling needle-assisted revision (NILLINR) to treat high-flow within the vascular access, which has the advantage of being safe, efficient, and time-saving compared to previous ones.

## Materials and methods

The Ethics Committee of Tongji Medical College, Huazhong University of Science and Technology approved this study (TJ-IRB20211119). The investigations conform to the principles outlined in the Declaration of Helsinki. All patients were informed of the nature of the study and consented to its specifics.

### Study populations

**Figure 1.**
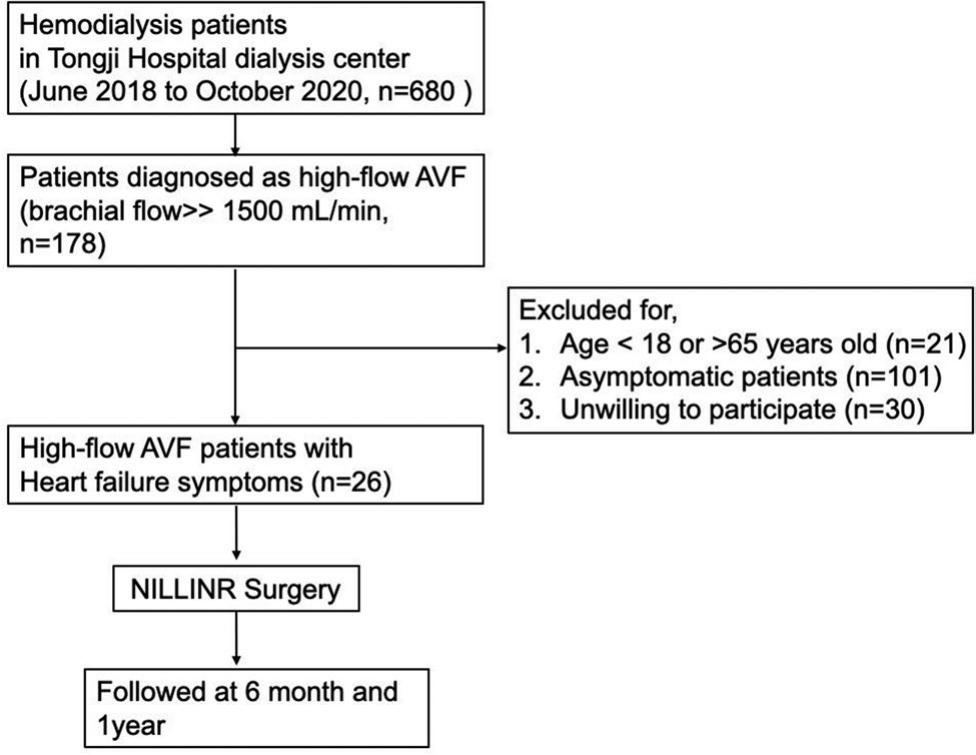
Flow chart of the study.

From June 2018 to October 2020, HD patients (*n* = 680) who attended their routine dialysis at Tongji Hospital dialysis center were screened. A total of 178 patients were diagnosed as high-flow AVF (Brachial artery flow > 1500 mL/min) and included for further selection [7]. Exclusive criteria were age < 18 years old, unwilling to participate, being asymptomatic (patients without heart failure symptoms, including shortness of breath with activity or when lying down fatigue and weakness, swelling in the legs, ankles, and feet, etc.). Patients aged > 65 years old were also excluded because of the difficult to rule out aging-related heart disease. Finally, we performed NILLINR surgery for 26 patients with comorbid heart failure and high-flow AVF. The flow chart of the study was clarified in Figure 1. The brachial artery flow before and after surgery was measured by experienced clinicians using the duplex Doppler ultrasound (DUS). All 26 patients were followed up for up to 1 year, and the brachial artery flow of participants was recorded before 6 months, and 1 year after surgery.

### The operation process of NILLINR

**Figure 2.**
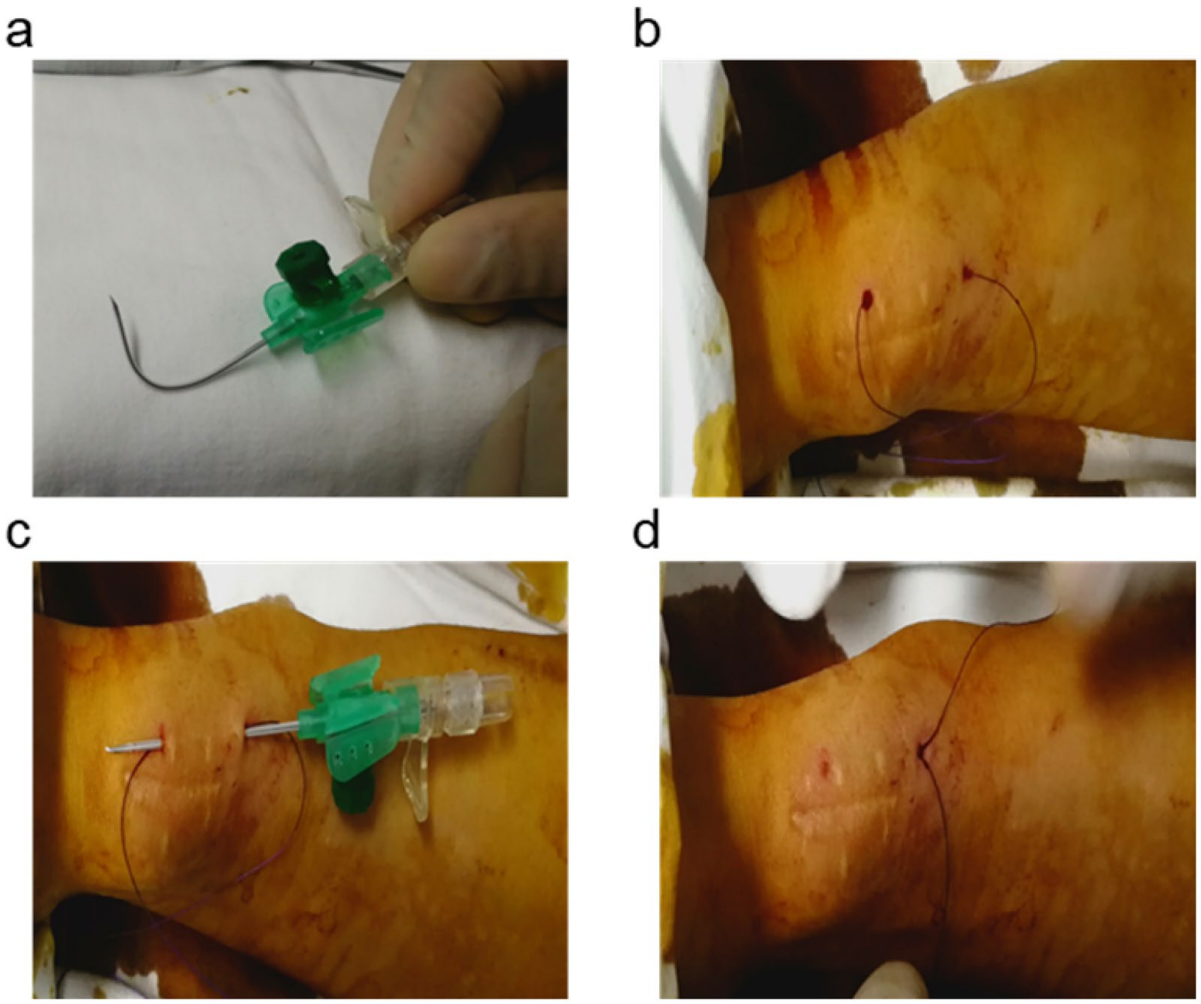
The operation process of NILLINR.

The whole procedure was guided with the duplex Doppler ultrasound (DUS). A surgical suture (Ethicon Vicryl, 2-0 size) was used with an indwelling needle (BRAUN Germany, 18 G) to traverse the arteriovenous anastomosis. DUS was performed to facilitate visualization of the needle and to determine brachial artery flow. Firstly, 2 indwelling needles were prepared, one with the original shape and the other bent for standby (Figure 2(a)). The curved indwelling needle passed through the bottom of the vessel proximity to the anastomosis under the guidance of ultrasound, and then the needle was pulled out to let the surgical suture reach the other side through the sheath (Figure 2(b)). The other indwelling needle was used to bypass the thread above the anastomosis (Figure 2(c)). Finally, the brachial artery flow was monitored by DUS, and the thread was knotted subcutaneously until the appropriate artery flow was reached (600-800 mL/min) (Figure 2(d)). The ultrasonic performance of the operation process is shown in Video S1.

## Results

In this study, 26 hemodialysis patients (20 males and 6 females; mean age 37.9 ± 10.8 years) with high-flow access were treated with NILLINR. The characteristics of patients and cardiac function evaluation are shown in Tables 1, 2 2. The establishment time of AVF ranges from 12 months to 56 months. 20 patients used radial-cephalic AVF as dialysis access.

**Table 1.** Clinical characteristics of patients with high-flow access.

Characteristics	
Age, mean ± SD, year	37.9 ± 10.8
Male, *n* (%)	20 (76.9%)
Diabetes, *n* (%)	9 (34.6%)
Age of AVF, mean ± SD, months	30.3 ± 11.2
AVF type	
Brachial-cephalic AVF, *n* (%)	6 (23.1%)
Radial-cephalic AVF, *n* (%)	20 (76.9%)

SD: standard deviation; AVF: arteriovenous fistula.

**Table 2.** Cardiac function evaluation of patients who underwent NILLINR procedures according to NYHA classification of heart failure.

Before operation	N (%)
Heart Failure	26 (100.0%)
NYHA classification	
I	0 (0.0%)
II	16 (51.5%)
III	10 (48.5%)
IV	0 (0.0%)
After operation	N (%)
Heart Failure	5 (19.2%)
NYHA classification	
I	21 (80.8%)
II	5 (19.2%)
III	0 (48.5%)
IV	0 (0.0%)

NYHA: New York Heart Association.

**Figure 3.**
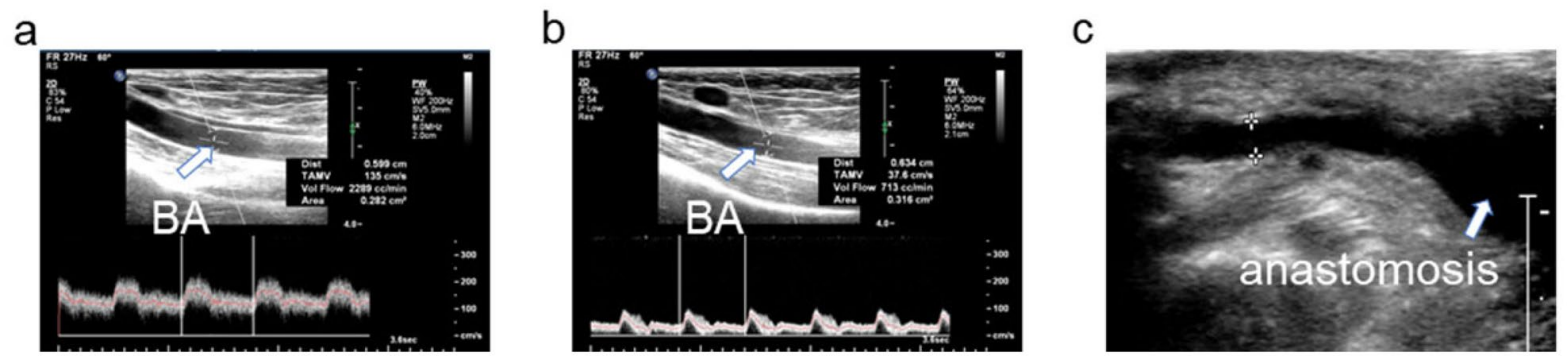
The changes in volume flow of the brachial artery before (a) and after (b) NILLINR operation. BA: brachial artery.

**Figure 4.**
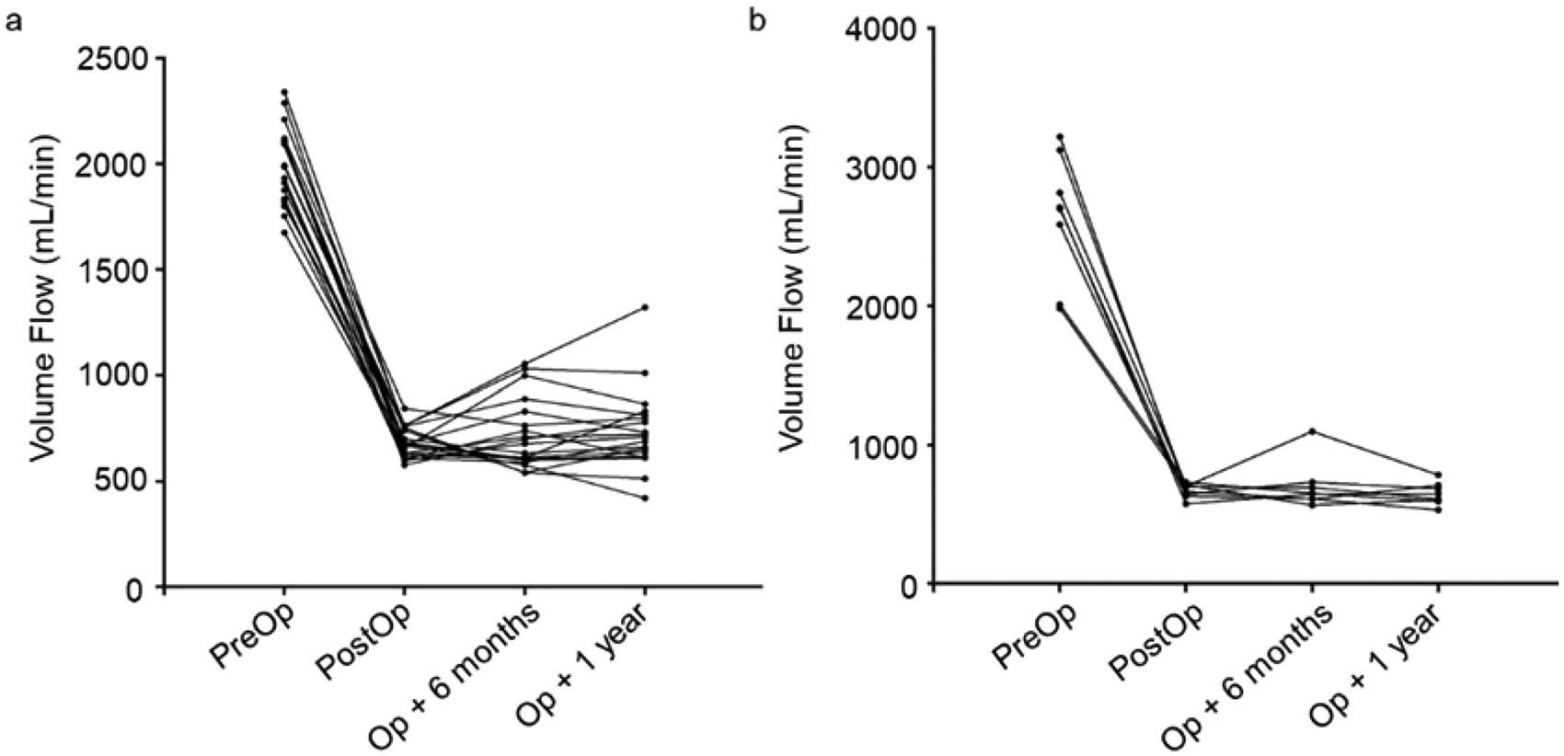
The changes in volume flow of the brachial artery before and after ultrasound-guided NILLINER procedure. (a) 1 banding procedure; (b) 2 banding procedure. PreOp, pre-operation; PostOp: post-operation; Op + 6 months: 6 months after operation; Op + 1 year: 1 year after the operation.

**Figure 5.**
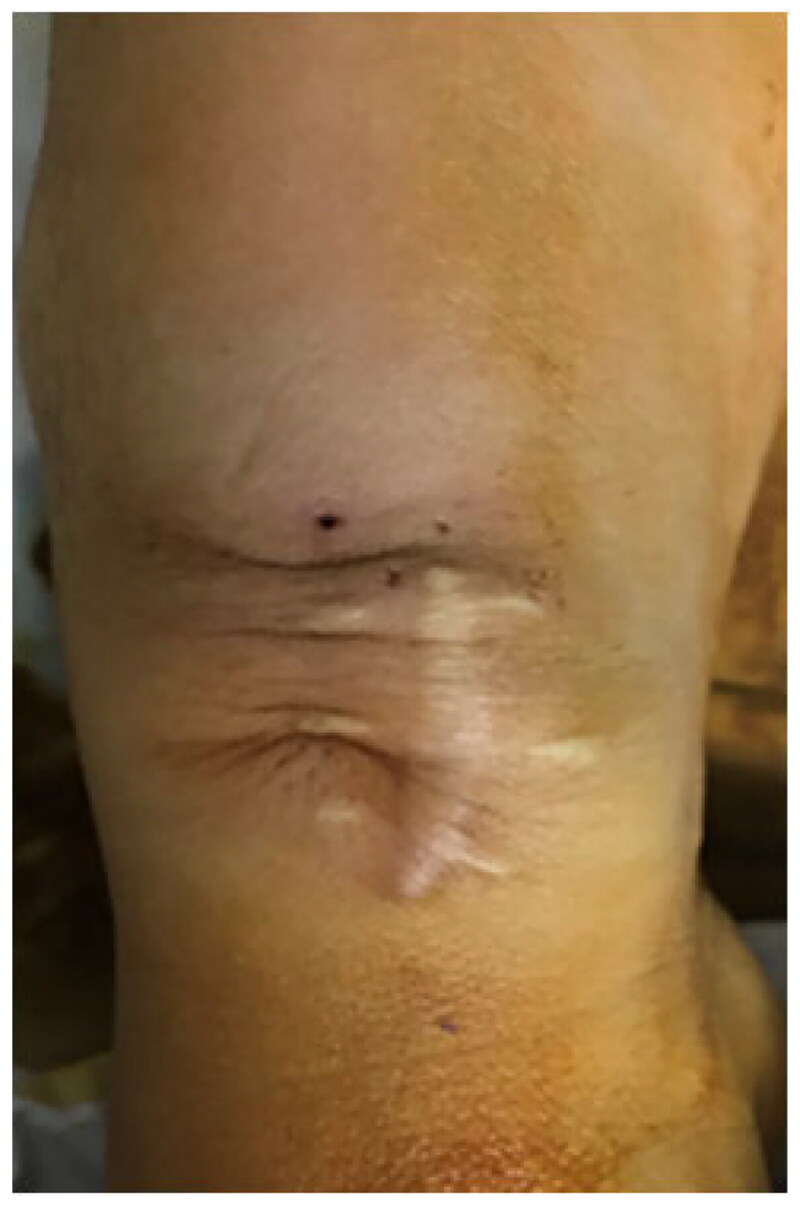
A representative picture shows the skin condition of the patient’s surgical site.

During the study period, 26 patients underwent a total of 34 NILLINR procedures (8 patients underwent 2 banding operations, and the remaining 18 patients underwent only 1 banding procedure). The 8 patients who needed a second procedure included 5 patients using brachial-cephalic AVF. After the operation, symptoms related to heart failure improved immediately after the operation in all patients (Table 2). The brachial artery flow before and after the operation of all patients is displayed in Table 3. After surgery, the patient’s brachial artery flow decreased (Figure 3). The volume flow ranged from 1675 to 3219 (mean ± SD, 2196.2 ± 416.9) mL/min before the operation and decreased from 2196.2 ± 416.9 mL/min to 679.2 ± 67.1 mL/min after the operation. In addition, the operation process was time-saving (mean ± SD, 8.5 ± 3.3 min), which ranged from 5 min to 15 min. During follow-up, we found that the volume flow of the brachial artery remained within the restricted range at 6 months (mean ± SD, 720.2 ± 164.7 mL/min, ranges from 541 to 1098 mL/min) and 1 year (mean ± SD, 713.9 ± 173.8 mL/min, ranges from 421 to 1322 mL/min) after the operation (Figure 4). It was also worth mentioning that all these AVFs were still in use for HD at 6 and 12 months. Compared with traditional open surgery and the MILLER procedure, this novel operation is less invasive (Figure 5). Besides, no adverse event of bleeding and vascular rupture was observed during the surgery procedure.

**Table 3.** Preoperative and postoperative brachial artery flow* of patients.

Variables	
Banding times	
1, *n* (%)	18 (69.2%)
2, *n* (%)	8 (30.8%)
Operation duration, mean ± SD, min	8.5 ± 3.3
The volume flow of the brachial artery before the operation, mL/min	2196.2 ± 416.9
The volume flow of the brachial artery post-operation, mL/min	679.2 ± 67.1
The volume flow of the brachial artery at 6 months post-operation, mL/min	720.2 ± 164.7
The volume flow of the brachial artery at 12 months post-operation, mL/min	713.9 ± 173.8

SD: standard deviation. *measured by duplex Doppler ultrasound.

## Discussion

The low-resistance venous outflow tract and appropriate pressure gradient of AVF are the keys to ensuring hemodialysis efficiency [7]. The flow of AVF is recommended to be maintained between 600 and 1500 mL/min [9]. On the one hand, the low flow is associated with compromised maturation of autologous AVF [10]. On the other hand, the establishment of AVF can lead to higher venous reflux with resultant high-output heart failure [11,12]. Choi’s team found that high-flow AVF was associated with an increased risk of myocardial fibrosis and heart failure in MHD patients [13]. Besides, it also affects pulmonary circulation and eventually induces pulmonary hypertension, which is another life-threatening complication for end-stage renal disease (ESRD) patients [14]. High-flow access is an easily unnoticed complication, which is usually caused by hypertrophy and low resistance of the outflow tract. For the blood vessels at the fistula, high-flow access may lead to lumen sclerosis and aneurysmal expansion in some cases [15]. In addition, the high flow of AVF sometimes induces DASS, which is mainly manifested as ipsilateral ischemia after the establishment of vascular access in maintenance dialysis patients [16].

At present, MILLER and some other traditional surgical banding operations are available methods for the treatment of high flow in AVF. Traditional surgical methods include distal revascularization-interval ligation (DRIL), revascularization using distal inflow (RUDI), and open surgery [17]. In the operation of DRIL, the artery is ligated at the distal end of the anastomosis, and then a bypass is established between the proximal artery and the native artery at the distal end of the ligation [16]. Compared with DRIL surgery, RUDI retains the original circulation mode which involves ligation of the native fistula and relocation of AV anastomosis onto a smaller artery [18]. In addition, open surgery is designed to increase vascular resistance by reducing the diameter of the fistula outflow tract. Fortunately, the above-mentioned ways can improve symptoms associated with high-flow vascular access. However, MILLER is less trauma and fewer complications [17]. MILLER not only benefits patients with DASS but also alleviates the clinical symptoms of high-flow patients [7,16]. In all our operations, the flow of the brachial artery is effectively restricted in most patients after a single banding procedure. No thrombosis and access stenosis were observed duration of the operation. Even in patients who did not have successful flow restriction after one banding procedure, clinical success was achieved after the second banding process. In addition, these patients’ vascular access flow remained within a reasonable range even after long-term follow-up. Significantly, the tool we need in this novel way is the indwelling needle instead of a balloon, which greatly reduces the economic burden of patients. Furthermore, there is no obvious incision after the banding operation, which reduces the risk of infection. The patients do not have to remove the suture after discharge.

Several limitations to this study need to be acknowledged. It is essentially a retrospective study with a small size. Furthermore, since all included operations were conducted in Chinese patients, these findings cannot be generalized to a wider population. In addition, as all included populations were relatively young, our results may not be generalized to the elderly population. However, this novel no-incision banding operation is safer and more efficient compared with other alternatives.

In conclusion, the no-incision limited ligation indwelling needle-assisted revision is a relatively safe, convenient, and available method for treating high-flow access in dialysis patients.

## Supplementary Material

Supplemental Material

## Data Availability

The data underlying this article will be shared on reasonable request with the corresponding authors.
